# Investigation of the Crystallization Characteristics of Intermediate States in Ge_2_Sb_2_Te_5_ Thin Films Induced by Nanosecond Multi-Pulsed Laser Irradiation

**DOI:** 10.3390/nano12030536

**Published:** 2022-02-04

**Authors:** Jia Du, Jun Zhou, Lianzhen Zhang, Na Yang, Xin Ding, Jin Zhang

**Affiliations:** Key Laboratory of Optical Technology and Instrument for Medicine, Ministry of Education, University of Shanghai for Science and Technology, Shanghai 200093, China; jz361@outlook.com (J.Z.); zlz16678188128@163.com (L.Z.); 191550058@st.usst.edu.cn (N.Y.); 201440055@st.usst.edu.cn (X.D.); 192540371@st.usst.edu.cn (J.Z.)

**Keywords:** Ge_2_Sb_2_Te_5_, laser-induced crystallization, multi-level intermediate states, Raman spectra, thermal simulations

## Abstract

Laser pulses can be utilized to induce intermediate states of phase change materials between amorphous and crystalline phases, making phase change materials attractive and applicable for multi-level storage applications. In this paper, intermediate states of Ge_2_Sb_2_Te_5_ thin films induced via employing a nanosecond multi-pulse laser with different energy and pulse duration were performed by Raman spectroscopy, reflection measurement and thermal simulations. Upon laser-crystallized Ge_2_Sb_2_Te_5_ films, optical functions change drastically, leading to distinguishable reflectivity contrasts of intermediate states between amorphous and crystalline phases due to different crystallinity. The changes in optical intensity for laser-crystallized Ge_2_Sb_2_Te_5_ are also accompanied by micro-structure evolution, since high-energy and longer pulses result in higher-level intermediate states (corresponding to high reflection intensity) and largely contribute to the formation of stronger Raman peaks. By employing thermal analysis, we further demonstrated that the variations of both laser fluence and pulse duration play decisive roles in the degree of crystallinity of Ge_2_Sb_2_Te_5_ films. Laser fluence is mainly responsible for the variations in crystallization temperature, while the varying pulse duration has a great impact on the crystallization time. The present study offers a deeper understanding of the crystallization characteristic of phase change material Ge_2_Sb_2_Te_5_.

## 1. Introduction

Chalcogenide phase change materials (PCMs) are a class of materials which can be repeatedly switched between amorphous and crystalline phases, typically by optical or electrical pulses. Such materials have significant applications because there are large differences in electrical and optical properties between these phases. Up to now, PCMs have been widely used in all-photonic memories [1,2,3,4,5,6], color display [7,8], neuro-inspired computing [9], reconfigurable meta-optics [10,11], photonic switches and routers [12,13], and so on. Among these PCMs, Ge_2_Sb_2_Te_5_ (GST) has received considerable attention in terms of its fast phase switching speed, high optical reflectivity, and outstanding scalability [1,5,14]. Different pulse duration time and energy of pulsed laser have a great influence on the material properties [1,15,16,17]. Conventionally, the amorphization process can be caused by a short high-power pulse, since it normally requires ultrafast melting and subsequent rapid quenching, whereas a relatively lower-power and longer pulse (namely, a continuous thermal process which above the glass-transition temperature) can result in crystallization. The rapid and reversible switching of conventional PCMs between amorphous and crystalline phases have two-level data storage, however, which greatly hinder the improvement of storage density and the complex encoding transfer [18,19]. In contrast, the reversible phase switching and excellent reflectivity contrast of GST via Joule heating are of great significance for allowing fabrication in multi-level storage, and the capacity can be improved greatly compared with two-level data storage.

A considerable number of investigations have focused on the laser crystallization characteristics of GST materials, since the understanding of the laser-induced crystallization process is crucial for the precise control of data reading and erasing. For instance, Weidenhof et al. [20] found that the irradiation area and the mean depth of crystalline spots increased with the elongation of pulse length. Zhu et al. [21] observed that the average grain size increased with the increase of laser fluence. Kunkel et al. [22] further described how the formation and growth of new nuclei under laser irradiation were affected by the high cooling rate. Except for the crystalline and amorphous states, depending on different pulse duration, laser energy, repetition rate, and number of pulses of laser pulses, the laser-crystallized GST could end up with different intermediate states. Wang et al. [23] obtained different metastable phases, distinguished by their different Raman vibration modes, by adjusting the laser power. Fan et al. [24] realized five-level storage with accumulated laser pulse irradiations compared to single laser irradiation. Yang et al. [25] showed that the same area of a film that is repeatedly exposed through multiple laser pulses could enhance crystallization effect, resulting in multi-level crystallization. Sun et al. [26] achieved the metastable phase with the different laser fluence via the use of femtosecond and nanosecond single laser pulses with pulse durations of 500 fs and 20 ns, respectively. Although laser-induced multi-level intermediate states of GST films has attracted considerable attention, most of the studies have focused on modulating the degree of crystallization by controlling the heat accumulation effects of laser pulses with different laser fluence or pulse numbers. In contrast, pulse duration integrated with laser fluence were also important controllable factors for inducing multi-states of phase change films. The multi-level switching of GST thin films induced by carefully controlling the drive energy per unit area and pulse duration were realized in our previous work [27]. Liu et al. [28] developed a 3D finite element simulation to investigate the effects of laser fluence and pulse duration on the temperature field of GST films. In addition, the variation of the crystallization temperature and crystallization time, which are critical for the degree of crystallization of GST films, vary remarkably with laser fluence and pulse duration. Hence, the crystallization characteristics of intermediate states in GST films induced by laser irradiation with varying laser fluence and pulse duration still need to be studied.

In this work, we aimed to study intermediate states of GST films induced by a nanosecond multi-pulsed laser with different pulse duration and energy. The crystallization characteristics of intermediate states by laser irradiation were elucidated in detail by using Raman spectroscopy, reflection measurement and thermal simulations. The significant change in reflectivity of multi-level intermediate states of GST was ascribed to that of difference in crystallinity. The correlation between the Raman spectra intensity and the crystallinity of laser-induced intermediate states was further discussed. Furthermore, the effect of variation in crystallization temperature and crystallization time on intermediate states were simulated, and it was demonstrated that the laser fluence and pulse duration were important factors for determining the different crystallinity of laser-crystallized GST.

## 2. Methods

### 2.1. Experiment

The amorphous Ge_2_Sb_2_Te_5_ (a-GST) thin films were deposited by thermal evaporation on a single crystalline silicon(c-Si) substrate. A deposition rate of ~10 Å/s and a film thickness of about 35 nm were observed by a quartz crystal monitor at a base pressure of 1.2 × 10^−4^ Pa. The film exhibited a composition of Ge_22.504±1.164_Sb_23.032±1.365_Te_54.464±1.193_, which was determined via energy disperse spectroscopy. In a temperature-controlled heating stage (Instec.inc), the a-GST film in a nitrogen atmosphere was annealed at 200 °C for 30 min to produce a nanocrystalline face-centered cubic (FCC) phase, namely crystalline GST (c-GST).

**Figure 1:**
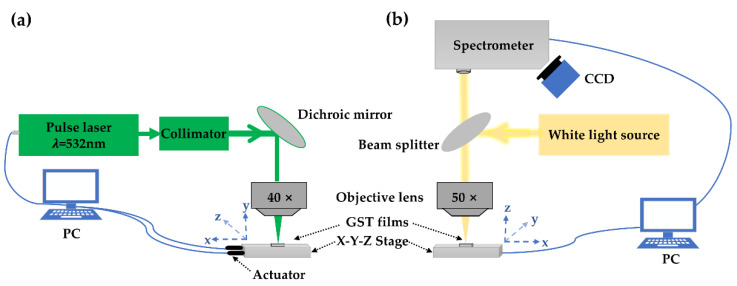
Schematic of the experimental set-up, (**a**) the 532 nm laser beam propagated along the green line for phase transition experiment with GST films; (**b**) the home-built micro-area reflection measurement system for GST films.

In order to realize phase switching of GST thin films with laser pulses, the 532 nm nanosecond pulsed laser with a repetition rate up to 100 kHz, pulse width varying from 5 to 250 ns and pulse number fixed at 10, was used as the pump laser, as shown in Figure 1a. The drive current of the laser varied from 0 to 10 A. Correspondingly, the output single pulse energy was changed from approximately 0 to 300 nJ (10 A & 5 ns). It is worth noting that the irradiation area still does not have any visible variation with the minimum two pulses for triggering crystallization, and the largest irradiation spot diameter with 10 pulses on the sample surface was about 12 μm. We used a home-built micro-area reflection measurement system (shown in Figure 1b) to obtain the reflectivity of the irradiation spots on GST films. The irradiation spot reflection spectra were collected by a spectrometer (iHR550 HORIBA). The reflection light source was a halogen tungsten lamp, and the probe light spot focusing on the sample surface had a diameter of about 5 μm. The position of the probe light spot on the sample was observed via a CCD camera.

The structural evolution was studied with a Raman spectrometer (XploRa Plus, HORIBA, France) equipped with 100× (0.9NA) microscopic objectives and a 638 nm-wavelength excitation laser. The spot size (related to the spatial resolution) of the focal plane in Raman measurements was about 0.864 μm. All spectra were recorded with a spectral resolution of 1 cm^−1^. In order to avoid laser-irradiated changes in the GST sample during continuous exposure, the power was kept below 3 mW. In addition, the irradiation topography was measured with a commercial AFM instrument (Nanosurf core AFM).

### 2.2. Simulation

To precisely characterize the crystallization process of phase change materials under the laser irradiation, the thermal evolution in the a-GST film on c-Si substrate was established by a finite element method by means of COMSOL Multiphysics. The heat transfer equation [29,30] can be given by:

(1) $$\nabla .(k(T)\nabla T)+Q=rho(T)c(T)\frac{\partial T}{\partial t}$$

where *c* is the specific heat, rho is the density, k is the thermal conductivity, T is the temperature, *t* is the time, and Q is the heat source of the laser beam and follows the Beer–Lambert law in irradiation depth distribution. The irradiation energy and the time evolution of the laser beam can be assumed to be a Gaussian distribution, as follows:

(2) Q(r,z,t)=P(r,t)(1−R(T))A(T)exp(−A(T)×z)

and

(3) $$P(r,t)=P_{P}(\frac{2}{\pi r_{w}{}^{2}})\exp[-2{\left(\frac{r}{r_{w}}\right)}^{2}]\exp[-{\left(\frac{t-t_{0}}{\tau /2}\right)}^{2}]$$

respectively, where, A(T) is the material absorption coefficient, R(T) is the surface reflectivity, P(r,t) is the incident laser power (W/m^2^), and $P_{p}$ is the peak power, $r_{w}\;$ is the beam radius, $t_{0}\;$ is the time shift, and  τ is the pulse width. By approximation, *t* > 0 can be considered for starting the simulation, and the full width at half maximum (FWHM) of the laser pulses is τ (namely, $t_{0}$ =  τ). The material properties of c-Si [28,30] and GST (namely, a-GST and c-GST) [21,31,32] are listed in Table 1.

Optical reflectivity of a-GST films was measured to be about 49.4% at 532 nm wavelength and improved to about 57.4% after crystallization. Real-time measurement of material properties in temperature dependance was difficult in such a short time of phase transition. We finally adopted the material properties shown in Table 1 to simulate the heating process. Additionally, lateral heat transfer was negligible, because the irradiation spot (~12 μm) was significantly larger than the absorption depth (calculated by Equation (4) [21] to be about 16 nm). Moreover, the thermal conductivity of the GST was smaller than that of the c-Si. This means that the energy absorbed was mainly focused on the film surface. The initial temperature of boundary conditions was defined as close to room temperature (293.15 K). Here, simulation parameters such as pulse energy, pulse width, and pulse numbers, were consistent with the experimental part. Due to a lack of data, structural transformations (i.e., the phase change from a-GST to crystallized or melted states) resulting from the latent heat of the heating/cooling process were neglected.

(4) $$d=\frac{\lambda }{4\pi k}$$

where *λ* is the wavelength and k (k = 2.65 [27]) is the imaginary part of the refractive index.

## 3. Results and Discussion

### 3.1. Irradiation Region Characteristics

**Figure 2:**
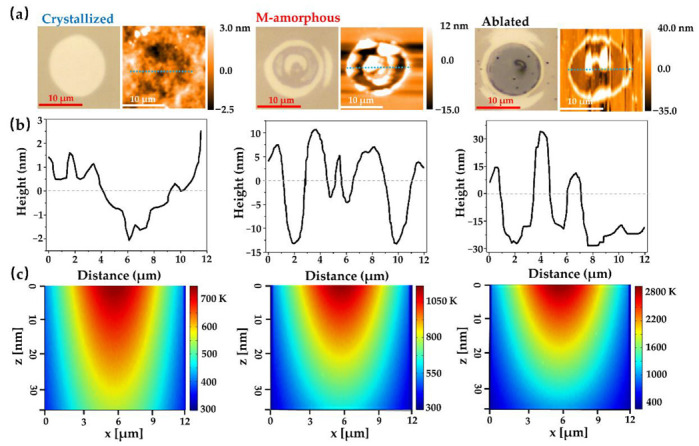
(**a**) Optical microscopy image (left) and AFM topography (right) of three categories of irradiation marks on the a-GST background film; (**b**) the heights distribution corresponding to cross-sectional views of blue dotted line in AFM topography; (**c**) thermal simulations of three categories of irradiation marks.

On the basis of our previous work, nanosecond-laser-induced regions on an a-GST background film can be defined as the crystallized, melt-quenched amorphous (M-amorphous) and ablated marks [27]. As shown in the optical images on the left panel of Figure 2a, the crystallized, M-amorphous and ablated marks on the a-GST background film are implemented by multi-pulse laser energy of 53.4 mJ/cm^2^ (&25 ns pulse width), 72.3 mJ/cm^2^ (&15 ns pulse width) and 88.9 mJ/cm (&5 ns pulse width), respectively. The topography evolution of three categories of irradiation marks characterized by using AFM is displayed in Figure 2a (right panel). It was reported that the density of a-GST film is remarkably increased after crystallization [33], thus resulting in the slight sinking of the crystallized region compared to the amorphous background. Note that, in the AFM surface images, the crystallized region is darker, while the amorphous background film displays the opposite. In addition, the AFM image emits noise during scanning measurement due to the larger roughness of the ablated regions. Figure 2b shows the cross-sectional views of the AFM topography (corresponding to the blue dotted line in Figure 2a), where the value represents the height distribution of the nanostructures. With regard to the crystallized mark at the laser fluence of 53.4 mJ/cm^2^ (&25 ns pulse width), the cross-sectional roughness was about 3 nm, indicating that the film was well crystallized. With a further increase of the laser fluence to 72.3 mJ/cm^2^ (&15 ns pulse width), the GST film was melted, and after the re-solidification process, the liquid quenching could result in a crystallized or locally and partially amorphized structure (evidenced by small toroidal dark areas in the AFM image). This phenomenon is probably due to the energy’s decaying outwards, which is caused by a relatively shallow melted depth, and the final structure after the quenching process was determined by the cooling rate, similar with results reported in [34]. Furthermore, increasing the laser fluence to reach 88.9 mJ/cm^2^ (&5 ns pulse width), the maximum removed depth was about 30 nm, which is close to the film thickness. It can be seen that the irradiation area was accompanied by a large number of the accumulation GST, which indicated the transition of films from normal evaporation to phase explosion due to a steep increase of the heating rate, similar to previously reported results [35]. This means that the topography distribution of the irradiation region was closely associated with the pulse duration and laser fluence.

Figure 2c shows the cross-sectional thermal field distribution of three categories of irradiation marks (corresponding to simulation time $t_{0}$) to illustrate the temperature variations of the irradiation regions more effectively. In general, the cross-sectional temperature distribution is above crystallization and melting temperatures that can refer to crystallized and M-amorphous states, respectively. The crystallization temperature of FCC phase (T_c_) is about 423 K and the melting point (T_m_) is near 894 K [28,36,37]. The melting interval of the GST material is estimated to have an upper limit of about 200–300 K above the equilibrium melting temperature due to the reduced activation energy of the melting phase, which is caused by overheating at super high heating rates [28]. For example, when the simulated temperature was less than the melting point but over the crystallization point, a large crystallization region was observed at the laser energy of 53.4 mJ/cm^2^ (&25 ns pulse width). By contrast, the temperature variations at laser fluences of 72.3 mJ/cm^2^ (&15 ns pulse width) were located in the melting interval, which was consistent with experimental observations. For high-energy and short pulses at laser fluences of 88.9 mJ/cm^2^ (&5 ns pulse width), the energy absorbed was mainly focused on the top surface center which was significantly higher than interior areas; thus, the steep increase of the heating rate led to GST evaporation or phase explosion.

**Figure 3:**
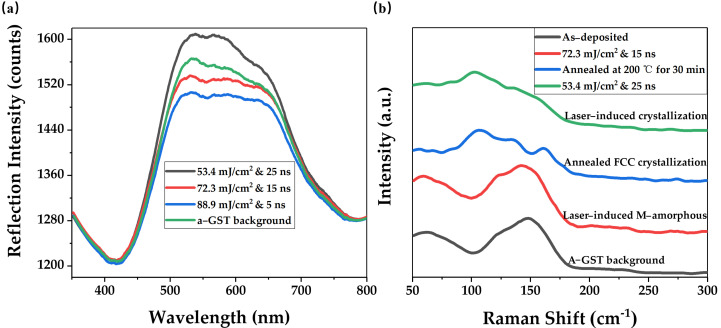
(**a**) Reflection intensity of three categories of irradiation marks on the a-GST background films; (**b**) Raman spectra characterization of M-amorphous and crystallized GST regions.

Three categories of irradiation marks were further characterized with reflection measurement and Raman spectroscopy. The transition between the crystalline phase and the amorphous phase changes the arrangement of the atomic structure in GST films, which leads to changes in optical properties, such as the complex refractive index. As shown in Figure 3a, the irradiation mark displayed a clear difference in reflection intensity. Apparently, the optical image of the crystallized mark was brighter, resulting in a reflection intensity greater than that of the amorphization state. Due to the GST’s evaporation in irradiation regions, which to some extent resulted from the high heating rate, the reflection intensity of the M-amorphous mark was lower than that of the a-GST background film. In addition, the ablated mark was darker under the optical microscope and its reflection intensity was lower, due to comparatively lower reflectivity of c-Si than that of GST film.

To further investigate the micro-structure evolution, we utilized Raman scattering measurements for irradiation marks, as shown in Figure 3b. The spectra of as-deposited GST (shown by the black line) had a large envelope peak at ~145 cm^−1^(related to Sb-Te vibrations in Sb*_m_*Te_3_ (*m* = 1, 2) pyramidal units) with a weaker peak at ~125 cm^−1^ (attributed to the vibration of A_1_ mode of GeTe_4–*n*_Ge*_n_*(*n* = 0, 1, 2) corner-sharing tetrahedra) [23,38,39]. Increasing the laser fluence reaching 53.4 mJ/cm^2^ (&25 ns pulse width), we could obtain the remarkable peaks of ~105 cm^−1^ and ~135 cm^−1^ (shown by green line) in the laser-crystallized mark, analogous to the Raman spectra of isothermally crystallized GST (shown by blue line) [39,40]. The former, at ~105 cm^−1^, was associated with the softened A_1_ modes of corner-sharing GeTe_4_ tetrahedra, and the latter, at ~135 cm^−1^, corresponded to the A_1_ mode of corner-sharing GeTe_4–*n*_Ge*_n_*(*n* = 1, 2) tetrahedra. However, isothermal annealing can re-arrange the atomic structures of the whole film in a long-enough time duration and steady environment, while laser pulses with a Gaussian distribution crystallize the GST in a local area in a very short time, thus leading to the laser-crystallized spectra with flat bands. Apparently, the peak at ~160 cm^−1^ (which corresponded to the vibration of the Sb*_m_*Te_3_ (*m* = 1, 2) subsystem) in the laser-induced crystallization spectra was very weak compared to the one crystallized by isothermal annealing. The results reveal that the GeTe component is mainly responsible for the phase transition under non-isothermal conditions, due to some extent to a certain similarity around Ge atoms in the structures of amorphous and laser-crystallized GST [23,41]. By increasing the laser energy to 72.3 mJ/cm^2^ (&15 ns pulse width), a remarkable broad peak at ~145 cm^−1^ with a shoulder at ~125 cm^−1^ was observed (shown by the red line). Note that the Raman spectra shown by the red line that derived from the laser irradiation were in accordance with the spectra of the as-deposited background films, verifying that the exposed irradiation area was melted and then fast frozen into an amorphization state again.

### 3.2. Laser-Induced Intermediate States

As analyzed above, we have characterized the structural evolution, topography, and thermal filed distribution of three categories of irradiation marks on the a-GST background film. In particular, the crystallized mark, which is induced by different laser pulses, can be composed of crystalline and amorphous GST materials in varying proportions. In this section, the effect of varying pulse duration and laser fluence on intermediate states of a-GST films was further investigated on the basis of simulations and experimental results.

**Figure 4:**
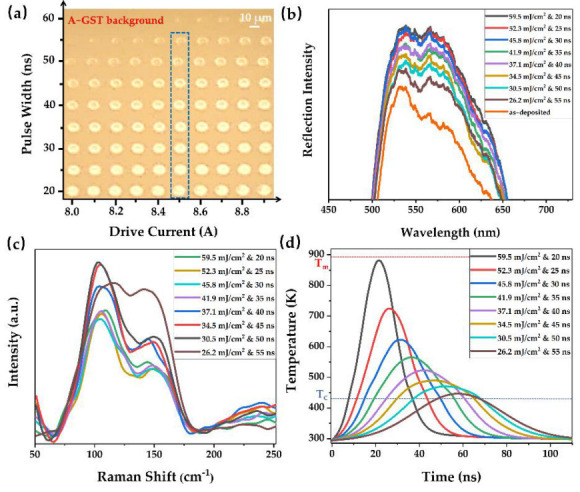
The characterization of multi-level intermediate states on an a-GST film induced by 10 laser pulses with varying drive current and pulse widths by (**a**) optical microscope image, (**b**) reflection spectra, (**c**) Raman spectra and (**d**) thermal simulations.

Based on the varying laser fluence and pulse width that needed to be researched regarding laser–material interactions, the pulse number of the nanosecond laser was fixed at 10 with a repetition rate of 100 kHz, and the irradiation marks on a-GST background film were shown in Figure 4a. The pulse width was set from 20 to 60 ns as the drive current increased from 8.0 to 8.9 A, which resulted in laser fluence between approximately 20.9 and 68.2 mJ/cm^2^ (8 A & 60 ns—8.9 A & 20 ns). It was noted that the size and brightness of the irradiation marks gradually increased with the increase of laser energy. Figure 4b shows the reflection spectra of the corresponding irradiation marks (illustrated with the blue dotted box in Figure 4a). These intermediate states exhibit multiple optical properties (optical constants or reflectivity) due to the mixture of crystalline and amorphous phases with different ratios. With the increase of laser energy, the crystallinity, refractive index, and reflectivity of GST thin films gradually increase, which are typical phenomena of multi-level phase switching. This means that the significant reflectivity changes, as well as the phase change, happened after the laser irradiation; accordingly, the high crystallinity of intermediate states corresponded to high reflectivity.

The Raman spectra of crystallized marks shown by the blue dotted box in Figure 4a were illustrated in Figure 4c. In the case of the laser fluence at 26.2 mJ/cm^2^ (&55 ns pulse width), there was no significant difference between the intensity of Raman peaks at ~105 cm^−1^ and ~135 cm^−1^. On the contrary, as the laser fluence was gradually increased, the dominated peak shifted to ~105 cm^−1^ with a weaker one at ~135 cm^−1^. Interestingly, the peak at ~105 cm^−1^ was considered as a characteristic peak of crystallization [42]. Among these intermediate states, the peak intensity at ~105 cm^−1^ was gradually increased with the increase of laser energy. The micro-structural changes in the GST lattice shown in Raman scattering, namely, peak intensity variation, could be related to locally and partially crystallized states of GST, which resulted from the ordering of defects and vacancies in the material [43]. A higher-level intermediate state along with high crystallinity, which was induced by a nanosecond laser with high-energy and longer pulses, could lead to stronger Raman peaks, due to the larger grains [21]. This signifies that the crystallinity of intermediate states may be associated with the peak intensity at ~105 cm^−1^.

In order to more effectively illustrate the laser-induced intermediate states, the maximum temperature variation at the top surface center of the above-mentioned irradiation marks was simulated, as shown in Figure 4d. The predicted maximum temperature at the top surface center was reached slightly later than that of the laser intensity peak ($t_{0}$) owing to thermal conduction. The rough transformation temperatures (namely, T_c_) and melting point (namely, T_m_) were marked by dashed lines. Apparently, the temperature variation trends, which are determined by the pulse width and laser fluence, could be largely attributed to the degree of crystallinity in laser-crystallized GST. For the a-GST material, crystallization occurs at a heating temperature below than the melting point but higher than the crystallization point. The high crystallization temperature and long thermal process could be observed by thermal simulations at relatively high-power and longer pulses, and it can be inferred that GST materials are accompanied by higher crystallinity with the elongation of the crystallization time. A good agreement of laser-induced crystallization between the simulated and experimental results was verified. Thus, we can further observe laser-induced intermediate states from the perspective of crystallization temperature and crystallization time through thermal simulations.

**Figure 5:**
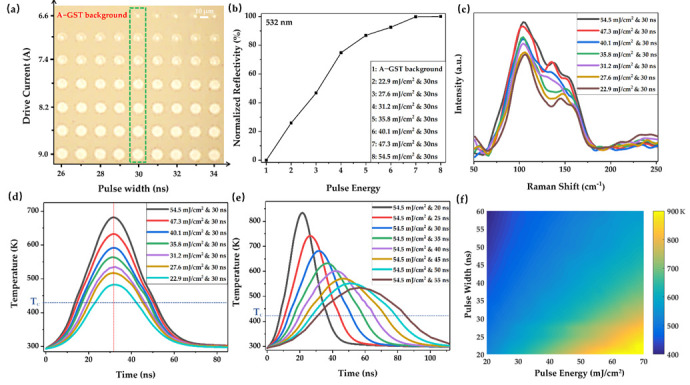
The irradiation marks on an a-GST background film characterized by (**a**) optical microscope image, (**b**) reflection spectra (normalized reflectivity), (**c**) Raman spectra and (**d**) thermal simulations; (**e**) thermal simulations for the maximum temperature variation at the top surface center of GST films induced with a certain laser fluence of 54.5 mJ/cm^2^ and varying pulse widths; (**f**) maximum temperature variations at the top surface center simulated by varying pulse energy and pulse widths.

The effect of varying laser fluence at a certain pulse width on the crystallization characteristics of GST was further investigated in Figure 5. The irradiation marks induced by nanosecond laser pulses with pulse widths varying from 26 to 34 ns were shown in Figure 5a. When phase switching at a certain pulse width, the drive current was varied from 6.6 to 9 A with step intervals of 0.4 A to observe the thermal effect at different laser fluences. Overall, the laser fluence was approximately varied from 20.5 to 56.8 mJ/cm^2^ (6.6 A & 34 ns~9 A & 26 ns). The highest reflection intensity is near 532 nm of reflection spectra due to the difference in absorption of the irradiation regions. The normalized reflectivity at 532 nm wavelength of irradiation marks (marked with the green dotted box in Figure 5a) was extracted and plotted in Figure 5b. With the laser fluence reaching 22.9 mJ/cm^2^ (&30 ns pulse width) and higher, the reflectivity of irradiation marks was gradually increased and was significantly higher than that of the a-GST background film, thus inducing intermediate states with higher crystallinity. It is widely known that the degree of crystallinity of laser-crystallized GST is related to the change in lattice structures, and a linear relation between the increase in reflectivity and the crystallinity for GST films has been reported [44]. Examination of Raman scattering was further adopted as shown in Figure 5c, where the structural change in the laser-crystallized GST film was evident with increasing laser energy. In laser-induced multi-level switching, as the laser fluence increased, the intensity of the Raman peak at ~105 cm^−1^ (which derived from the vibration of GeTe_4–*n*_Ge*_n_*(*n* = 0) tetrahedra) gradually increased, and this intensity is significantly stronger than that of the peak at ~135 cm^−1^. More specifically, more Te-rich structures tend to form in higher-level crystallization due to the laser-heating effect, as reported in our previous investigation [27]. A similar micro-phenomenon was also observed in [24], according to which the dominant characteristic peaks were sensitive to the size of the nanocrystal in GST films with increased laser pulse irradiations. In addition, the increase in crystallinity of the intermediate states corresponds with the increase of laser energy, since the columnar grains became larger with the increase of laser fluence observed by transmission electron microscopy in reference [21]. Figure 5d shows the maximum temperature variation at the top surface center of the irradiation marks shown with the green dotted box in Figure 5a. While the laser pulse width was fixed at 30 ns, the heating and cooling rates gradually increased as the laser fluence increased. However, the predicted maximum temperature at the top surface center appears at 32 ns, depicted by the red dashed line, which is slightly later than that of the laser intensity peak (30 ns) due to thermal conduction. Moreover, the temperature rise was mainly concentrated in the first several nanoseconds; as a result, a high crystallization temperature was generated due to the large heating rate under relatively high laser fluence. In other words, under the same pulse duration, the maximum crystallization temperature and total crystallization time varied with the laser fluence. In addition, the cooling process of the phase transformations of the GST films induced by nanosecond laser pulses had a great effect on crystallization as reported by [22,28,34]. The temperature variation at the top surface center of GST induced by laser pulse with pulse width varying from 20 to 55 ns at the laser fluence of 54.5 mJ/cm^2^ was further investigated, as shown in Figure 5e. It was observed that the maximum simulated temperature gradually decreases with the increase of pulse widths, and the cooling rate accordingly becomes slower, however, resulting in the elongation of crystallization time. It can be seen that the cooling process is mainly influenced by the pulse duration. However, such factors as the maximum temperature and maximum heating/cooling rates also vary remarkably with pulse duration at a certain laser fluence. That is, the laser fluence determines the maximum heating rate and is mainly responsible for the variation in the crystallization temperature of the irradiation regions, while the pulse duration contributes largely to the cooling rate and the crystallization time of the GST material.

In the following, the predicted maximum temperature at the top surface center of the laser irradiation areas versus the pulse energy and the pulse width is mapped, as shown in Figure 5f. Through different combinations of pulse energy and pulse width, this effectively illustrates the different crystallinity of GST with temperature dependance. These intermediate states of incomplete crystallization mainly result from a lower temperature or shorter temporal duration during crystallization. More importantly, high-level intermediate states of GST can result from high laser energy, along with relatively long pulse width, since the crystallization process typically requires tens to hundreds of nanoseconds for the nuclei to form and grow. However, the changes to the nano- and micro-structures of the GST materials are quite complicated. The quantitative analysis in the crystallinity of laser-crystallized intermediate states should be further studied with different characterization techniques such as the electron backscattering diffraction and transmission electron microscopy on the basis of systematic investigations of the temperature field.

## 4. Conclusions

In conclusion, we have investigated the crystallization characteristics of intermediate states in a-GST thin films induced by a 532 nm nanosecond multi-pulse laser with different pulse duration and energy. Through reflection measurement and Raman scattering spectra, it is demonstrated that laser-induced intermediate states are generated, while significant changes in the reflection intensity of intermediate states are the result of the difference in crystallinity of GST’s composed of different crystalline and amorphous molecules. It is observed that higher-level intermediate states are related to high reflection intensity, which is induced by a nanosecond laser with high-energy and longer pulses. This tends to form a stronger Raman peak at ~105 cm^−1^ with a weaker one at ~135 cm^−^^1^. In addition, on the basis of the experimental results, a thermal simulation is established, indicating that the variation in both laser fluence and pulse duration contribute to the degree of crystallinity of the intermediate states. The former could determine the crystallization temperature and heating rate, while the crystallization time and cooling rate mainly depend on the effect of pulse duration. The analysis of the crystallization characteristics of laser-induced intermediate states in GST thin films can lay the foundation for various future multi-level devices.

## Figures and Tables

**Table 1:** The material properties of c-Si and GST.

Material	Density (kg/m^3^)	Thermal Conductivity (W/m∙K)	Specific Heat (J/kg∙K)
a-GST	5860	0.25	228
c-GST	6130	0.45 ± 0.09	240
c-Si	2330	140	700

## Data Availability

Data sharing not applicable.
